# Navigator readouts with optimized processing improve spinal cord and gray matter segmentability in multi-echo gradient-echo imaging

**DOI:** 10.1162/IMAG.a.1320

**Published:** 2026-08-03

**Authors:** Silvan Büeler, Laura Beghini, Christian W. Kündig, Martina D. Liechti, S. Johanna Vannesjo, Gergely David

**Affiliations:** Department of Neuro-Urology, Balgrist University Hospital, University of Zurich, Zurich, Switzerland; Spinal Cord Injury Center, Balgrist University Hospital, University of Zurich, Zurich, Switzerland; Department of Physics, Norwegian University of Science and Technology, Trondheim, Norway

**Keywords:** navigator correction, B_0_ field fluctuations, breathing, spinal cord, gray matter, segmentation

## Abstract

Multi-echo gradient-echo (ME-GRE) acquisitions of the spinal cord (SC) are highly susceptible to breathing-induced magnetic field fluctuations arising from the proximity of the lungs, often resulting in ghosting artifacts and signal loss. A navigator readout can be employed to monitor and correct for these field variations; however, conventional navigator processing methods frequently fail in the SC due to its small size and distinct anatomy. Here, we evaluate the impact of a recently introduced, SC-specific navigator processing approach on SC and gray matter (GM) segmentability. ME-GRE images were acquired at 3T during free breathing in ten healthy volunteers, covering the cervical, thoracic, and lumbosacral spinal cord. Data were collected both with and without a centerline navigator readout. Acquisitions without navigators included five imaging echoes, whereas those with navigators comprised four imaging echoes followed by an additional readout of the k-space centerline (the “navigator echo”). The navigator readout was processed using a recently introduced method optimized for the SC. The effect of the optimized navigator correction was evaluated by contrast-to-noise ratio (CNR), qualitative ratings of SC and GM segmentability, and the performance of automatic segmentation algorithms. Compared with acquisitions without navigators, optimized navigator correction significantly improved quantitative image quality metrics, particularly GM-white matter CNR at the C7-T1 and T7-T8 vertebral levels and across the lumbosacral cord. Regarding SC and GM segmentability, raters preferred images with navigators over uncorrected images at nearly all spinal cord levels, with the strongest effect observed for GM segmentability in the lumbosacral cord. Although overall automatic segmentation performance was comparable between acquisition types, automatic GM segmentation failed at the lumbosacral cord in the presence of severe artifacts in acquisitions without navigators, while remaining robust in the corresponding navigator-corrected images. In conclusion, navigator processing optimized for SC imaging improves image quality and segmentability in ME-GRE acquisitions, making this technique attractive for both clinical and research applications.

## 1 Introduction

Multi-echo gradient-echo (ME-GRE) sequences are widely used for spinal cord (SC) imaging due to their high contrast between gray matter (GM), white matter (WM), and cerebrospinal fluid (CSF) (Cohen-Adad et al., 2021; Stroman et al., 2014). They can be used to depict lesions in multiple sclerosis (White et al., 2011) and allow for cross-sectional area (CSA) measurements of the SC, GM, and WM. CSA has been used to assess atrophy in longitudinal studies of conditions such as spinal cord injury (David et al., 2021) and in studies with longitudinal clinical follow-up in amyotrophic lateral sclerosis (Paquin et al., 2018). ME-GRE sequences are also employed in quantitative techniques such as susceptibility mapping, relaxometry, and myelin water imaging (Wheeler-Kingshott et al., 2014).

However, ME-GRE sequences are highly sensitive to spatial and temporal fluctuations of the magnetic field (B_0_), which can cause ghosting artifacts and signal loss (Czervionke et al., 1988; Raj et al., 2000; Schenck, 1996). Local magnetic susceptibility differences between soft tissues and the lungs, together with respiratory-induced changes in lung volume, generate dynamic B_0_ fluctuations. Although similar effects occur in the brain (Versluis et al., 2010), they are particularly pronounced in the thorax and abdomen. Consequently, breathing-related field fluctuations remain a major source of artifacts in spinal cord imaging (Verma & Cohen-Adad, 2014), limiting image quality and quantitative accuracy.

In the brain, a common approach to correct for breathing-induced B_0_ fluctuations is the use of a navigator readout, which is often implemented as an additional central k-space line acquired after each excitation (De Crespigny et al., 1995; Durand et al., 2001; Versluis et al., 2010, 2012; Wallace et al., 2022). Navigator readouts enable direct measurement of B_0_ fluctuations, allowing compensation through signal demodulation, which stabilizes the phase of the acquired data and reduces artifact load. However, despite their potential to improve image quality, navigators are rarely used in spinal cord imaging. This is likely because spinal cord imaging poses additional challenges compared to brain imaging (Stroman et al., 2014). In our experience, standard navigator implementations frequently fail, sometimes even exacerbating artifacts (see Fig. 6 in Beghini et al., 2025). Recently, we developed an optimized processing pipeline for robust navigator-based retrospective correction of breathing-induced B_0_ field fluctuations in the spinal cord (Beghini et al., 2025). Compared to standard navigator processing, this approach improved image quality by reducing ghosting artifacts and increasing both contrast-to-noise (CNR) and signal-to-noise ratio (SNR). The improvement was particularly pronounced in the lower cervical/upper thoracic cord and the lumbosacral cord, as well as at echo times longer than 19 ms. Although image quality metrics such as CNR and SNR are important for image segmentation, tissue segmentability is also strongly influenced by other image properties, including boundary sharpness and signal homogeneity (Wang et al., 2016).

In this study, we build on previous work to investigate whether a navigator readout combined with optimized navigator-based correction improves SC and GM segmentability in ME-GRE acquisitions of the cervical, thoracic, and lumbosacral spinal cord at 3T, thereby increasing the clinical utility of these images. In addition to image quality metrics, SC and GM segmentability was evaluated qualitatively by experienced raters using segmentability ratings and quantitatively by assessing the performance of an automatic segmentation algorithm. We hypothesized that optimized navigator-based correction would improve both subjective segmentability and the robustness of automatic SC and GM segmentation, particularly in the cervicothoracic junction and the lumbosacral cord, which are strongly affected by breathing-induced B_0_ fluctuations.

## 2 Material and Methods

### 2.1 Participants and study design

Ten healthy volunteers (3 females, 7 males; age (mean ± standard deviation): 33.6 ± 10.3 years, range: 22-54 years; body mass index (BMI, mean ± standard deviation): 24.0 ± 3.2 kg/m^2^, range: 19.9–29.1 kg/m^2^) participated in this study. The study was approved by the local ethics committee (Kantonale Ethikkommission Zürich, BASEC ID: 2019-00074) and conducted in accordance with the Declaration of Helsinki. Written informed consent was obtained from all participants.

**Fig. 1.**
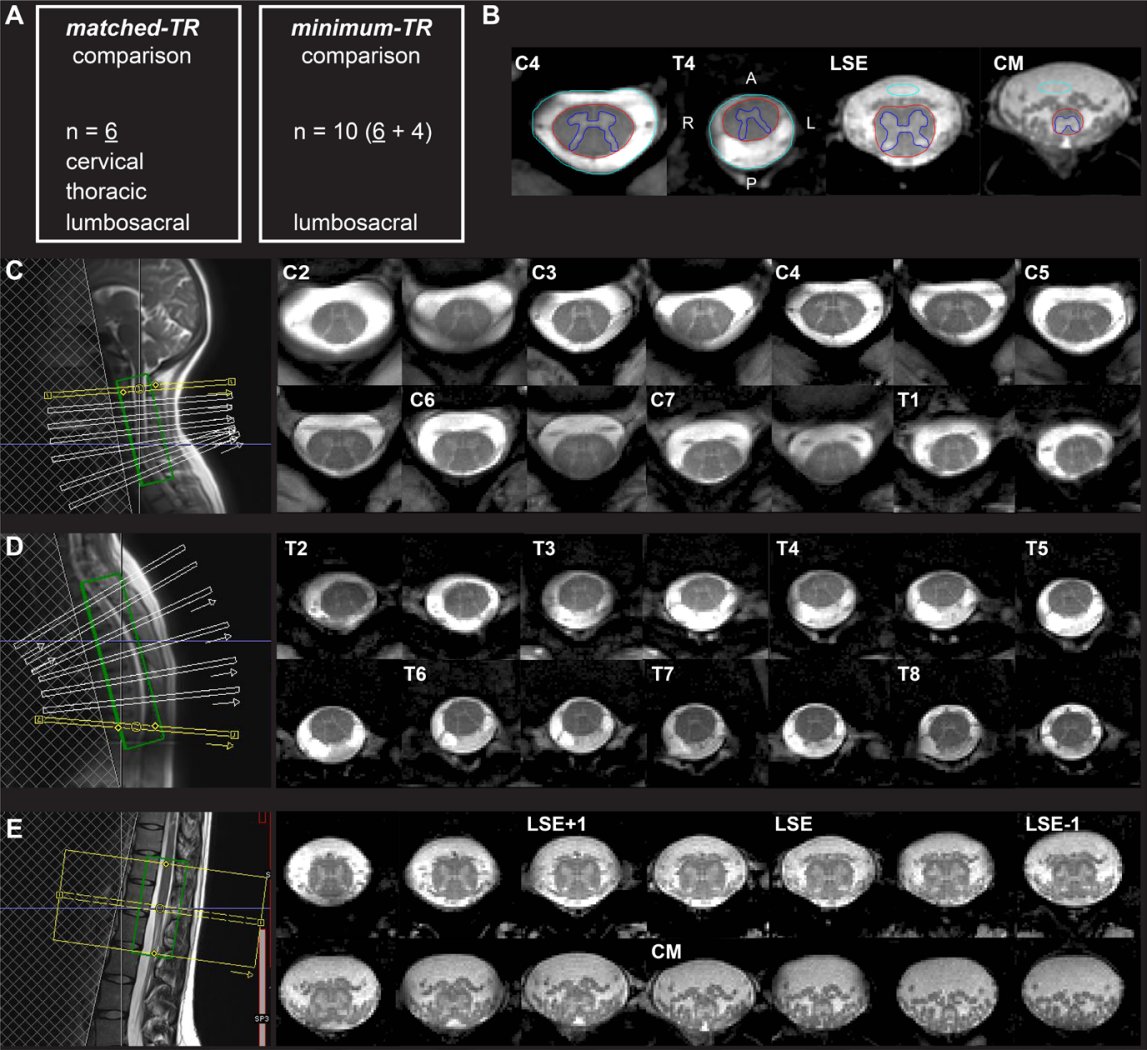
Image acquisition. (A) Study design. (B) Example of spinal cord (red outline), gray matter (blue outline), and cerebrospinal fluid (light blue outline) masks, used to compute image quality metrics, shown here at selected anatomical landmarks: vertebral levels C4 and T4, the lumbosacral enlargement (LSE), and the conus medullaris (CM). (C–E) Sagittal slice positioning (left panel) and corresponding axial slices of the 2D ME-GRE sequence (right panel) in the cervical (C), thoracic (D), and lumbosacral spinal cord (E). The shimming box, outlined in green, encompassed a volume that extended slightly across the spinal canal in the left–right (LR) and anterior–posterior (AP) direction. A saturation band was placed anterior to the vertebral column to suppress signals from the head, neck, thorax, and abdomen. Anatomical landmarks rostral (“LSE+1”, two slices rostral to the LSE) and caudal (“LSE-1”, two slices caudal to the LSE) relative to the LSE are also indicated. The axial slices shown were acquired with navigators.

The study consisted of two comparisons (Fig. 1A): In the *matched-TR* comparison, six participants (2 females, 4 males; age: 37.9 ± 12.0 years) underwent imaging of the cervical, thoracic, and lumbosacral spinal cord. For each region, 2D spoiled ME-GRE (Siemens FLASH) images were acquired both with and without navigators, using identical slice geometry and matching repetition times (TR). To minimize potential systematic bias, the acquisition order was counterbalanced across participants with respect to both acquisition type (with vs. without navigators) and spinal cord region (cervical, thoracic, and lumbosacral). Within individual participants, however, the sequence order was kept consistent across regions; for example, participants who underwent the navigator sequence first did so in all regions, followed by the sequence without navigators (or vice versa).

In the *minimum-TR* comparison, a larger cohort of ten participants (the original six plus four additional volunteers) underwent lumbosacral cord imaging only, as the largest quantitative differences between the sequences with and without navigators had been observed in this region (Beghini et al., 2025). In clinical practice, the minimum achievable TR and various image filters are typically used. Therefore, the sequence without navigators was acquired using the minimum TR, and standard image filters (2D distortion correction, coil sensitivity correction (Siemens Prescan Normalize), and elliptical filtering). We note that the navigated sequence was already acquired at the minimum TR and was unchanged for this analysis.

### 2.2 Image acquisition

MRI data were acquired on a 3T Siemens Prisma scanner (Siemens Healthineers, Erlangen, Germany) using the integrated body transmit coil and two receive coils: the standard 32-channel spine coil and the 64-channel head and neck coil. Both receive coils were used for imaging the cervical and thoracic cord, while only the spine coil was used for the lumbosacral cord. To minimize motion artifacts, participants were positioned according to standardized procedures as described in Büeler et al. (2022). These procedures included placing foam wedges under the knees to reduce lumbar lordosis, placing a vacuum cushion beneath the legs, and securing Velcro straps around the hips, knees, and chest. All imaging was performed during free breathing.

2D ME-GRE sequences (Siemens FLASH) with and without navigators were acquired in the cervical, thoracic, and lumbosacral cord. To acquire the sequence with navigators, the “Phase stabilization” option, available in the vendor-provided ME-GRE sequence (Syngo VE11C) but hidden by default, was enabled via the sequence programming environment. When enabled, this option acquired one central k-space line (along the left–right readout direction) after the last echo in each TR for each slice. The sequence also included the readout of a noise profile without signal excitation. We note that in our previous work, images without navigators were reconstructed from data acquired with navigators, allowing isolation of navigator effects but yielding a comparison that was not practically representative and disadvantaged the approach without navigators (Beghini et al., 2025). Notably, within the same acquisition time, a sequence without navigators can acquire five imaging echoes, whereas a sequence with navigators acquires four. Given that inclusion of a fifth echo improves SC segmentation (Büeler et al., 2022), we aimed to perform a more practically relevant comparison between optimized protocols, namely acquisitions with navigators with four echoes and acquisitions without navigators with five echoes.

For the cervical and thoracic spinal cord, imaging was performed using multi-slab sequences, where each of the seven slabs consisted of two axial-oblique slices (5 mm thickness, no gap), individually centered at mid-vertebral levels (C2–T1 and T2–T8, respectively). Slabs were oriented perpendicularly to the spinal cord to minimize partial volume effects (Fig. 1C, D). In the lumbosacral cord, due to the anatomical mismatch between vertebral and neurological levels, a single slab containing 18 axial-oblique slices (5 mm thickness, no gap) was acquired, also angulated perpendicularly to the spinal cord. Slab placement was guided by a sagittal T2-weighted turbo spin echo image to ensure full coverage of the lumbosacral enlargement (LSE) and conus medullaris (CM), following the approach described in Büeler et al. (2022) (Fig. 1E).

Detailed imaging parameters for both versions of the ME-GRE sequences (with and without navigators) are summarized in Table 1. Both sequence versions were acquired with identical geometry, resolution, echo spacing, and bandwidth. The sequence without navigators consisted of five echoes, whereas the sequence with navigators consisted of four echoes, followed by the k-space centerline navigator readout. The minimum achievable TR was slightly shorter for the sequence without navigators. In the *matched-TR* comparison, both sequence versions were acquired with identical TRs for each spinal cord region (cervical, thoracic, and lumbosacral) to isolate the effects of the navigators. In the *minimum-TR* comparison, performed in the lumbosacral cord only, the sequence without navigators was acquired at the minimum TR (878 ms vs. 899 ms in the *matched-TR* condition). The ME-GRE sequence in the spinal cord is typically acquired with more than one repetition to improve SNR (Cohen-Adad et al., 2021). In this study, three repetitions were acquired as a practical compromise between improved SNR and feasible acquisition time.

**Table 1.** Main sequence parameters for the 2D multi-echo gradient-echo sequences acquired with and without navigators.

	Lumbosacral		Cervical/thoracic	
	with nav.	w/o nav.	with nav.	w/o nav.
Varying parameters				
Echo time (ms)	6.86–18.86	6.86–22.86	6.86–18.86	6.86–22.86
Number of echoes	4 + 1 nav.	5	4 + 1 nav.	5
Repetition time (ms)	899	899 (*matched-TR*) 878 (*minimum-TR*)	700	700
Flip angle (°)	44	44	38	38
Number of slabs	1	1	7	7
Number of slices per slab	18	18	2	2
Acquisition time (min:sec)	9:14	9:12 (*matched-TR*) 8:59 (*minimum-TR*)	7:12	7:10
Image filters	no	no (*matched-TR*) standard filters* (*minimum-TR*)	no	no
Identical parameters				
Echo spacing (ms)	4			
Slice thickness (mm)	5			
FOV (mm^2^) read x phase (L-R x A-P)	192 x 192			
Resolution (mm^2^)	0.5 x 0.5			
PAT	GRAPPA 2x			
Bandwidth (Hz/pixel)	260			
Flow compensation	yes			
Number of repetitions	3			

A-P, anterior-posterior direction; FOV, field of view; GRAPPA, Generalized Autocalibrating Partial Parallel Acquisition; L-R, left-right direction; PAT, parallel acquisition technique.

* Standard filters consisted of an elliptical filter, 2D distortion correction, and coil sensitivity correction (Prescan Normalize).

In addition, in each region, a fully sampled, low-resolution GRE reference scan was acquired using the same slice geometry, with the following parameters: in-plane resolution of 2 × 2 mm^2^, in-plane field of view of 256 × 208 mm^2^, echo time of 3.06 ms, TR of 600 ms, flip angle of 25°, a single repetition, phase encoding along the posterior-anterior direction, and an acquisition time of 01:04 min.

### 2.3 Navigator-based correction and image reconstruction

The ME-GRE images acquired with navigators were reconstructed using the navigator processing pipeline described in detail in our previous publication (Beghini et al., 2025). Navigator corrections and image reconstructions were performed offline on the k-space data. The data were converted to the ISMRMRD format (Inati et al., 2017) and imported into Julia (Bezanson et al., 2017) using the MRIReco.jl package (Knopp & Grosser, 2021). The correction was performed using the MRINavigator.jl package (v0.1.1, https://github.com/NordicMRspine/MRINavigator.jl), which provides all functions required for navigator data extraction, multiple processing pipelines, and raw data correction. Among the navigator processing pipelines proposed by Beghini et al. (2025), the fast Fourier transform-based approach demonstrated the best performance and was therefore used in this study. It was specifically designed to provide accurate estimates of field fluctuations in the spinal cord region. The first step consisted of computing a one-dimensional fast Fourier transform of each navigator profile (De Crespigny et al., 1995; Versluis et al., 2010), yielding a phase-sensitive projection line along the frequency-encoding direction. Subsequently, an interval width of 3.5 cm centered on the spinal cord centerline was defined, and all samples outside this interval were discarded. Breathing-induced field fluctuations at each TR were estimated as the difference in phase relative to the first navigator readout, using SNR-weighted averaging across samples and coils. These field measurements were then used to remove the corresponding breathing-induced phase shifts from the acquired k-space data by demodulation.

Image reconstruction was performed in MRIReco.jl using a conjugate-gradient SENSE algorithm (10 iterations, L2 regularization) (Knopp & Grosser, 2021; Pruessmann et al., 1999). The sensitivity maps for the SENSE reconstruction were computed using the ESPIRiT algorithm (Uecker et al., 2014) implemented in MRIReco.jl, applied to the reference acquisition without masking. Masks for both the sensitivity maps and the final reconstructions were derived from the GRE reference images using MRINavigator.jl. A noise pre-whitening step was applied prior to reconstruction to reduce noise correlation across coils and thereby optimize SNR (Pruessmann et al., 2001).

For the images acquired without navigators, the standard online image reconstruction was used. Since the offline reconstruction of the images with navigators did not apply any filters, the ME-GRE images were also reconstructed on the scanner without filters for the *matched-TR* comparison. In contrast, for the *minimum-TR* comparison, standard image filters (2D distortion correction, coil sensitivity correction (Siemens Prescan Normalize), and elliptical filtering) were applied to mimic a typical clinical acquisition.

### 2.4 Image segmentation and motion correction

After reconstruction, root-mean-square (RMS) images over the first three, four, and five echoes were computed separately for each repetition and then averaged across repetitions. The SC, GM, and CSF were manually segmented by an experienced rater (S.B.) in JIM (v7.0; Xinapse systems Ltd, UK; http://www.xinapse.com), as described previously (Büeler et al., 2022, 2024), and were quality-checked by another experienced rater (G.D.). Segmentation was performed on the RMS images computed over the first three echoes of the image acquired with navigators and averaged across the three repetitions, as this approach has been shown to be optimal for joint SC and GM segmentation (Büeler et al., 2022). Manual segmentation was feasible across all slices, with high confidence for most.

The segmentations were then binarized using a threshold of 1 for the SC (i.e., only voxels fully contained within the segmentation were included to minimize partial volume effects with the CSF), and 0.5 for the GM. In the lumbosacral cord, binary WM masks were created by subtracting the binary GM masks from the SC masks. In the cervical and thoracic cord, the binary GM masks were dilated by one voxel before subtraction to minimize partial volume effects, given the small GM area in these regions. For the CSF mask, an ellipsoidal region of interest (area: 7.5 mm²) was placed anterior to the spinal cord in the lumbosacral cord (Fig. 1B). In the cervical and thoracic cord, the CSF mask was defined as the vertebral canal segmentation (binarized at a threshold of 0.5) minus the binary SC mask, followed by an additional one-voxel erosion.

To correct potential spatial misalignments among the three repetitions of an acquisition, each repetition was manually inspected and, if necessary, aligned slice-wise to the SC mask. Alignment was performed by shifting the images in-plane by an integer number of voxels, thereby avoiding interpolation-based resampling, which could slightly blur tissue boundaries and affect quantitative segmentation metrics. Whenever such an adjustment was required, a new average image was generated across repetitions. The same procedure was used to correct possible misalignments between images acquired with and without navigators. Overall, alignment was necessary for 64% of slices in the cervical cord, 8% in the thoracic cord, and 9% in the lumbosacral cord.

### 2.5 Signal- and contrast-to-noise ratio

Multiple image quality metrics were computed to compare images with and without navigators. The SNR was calculated for GM and WM as SNR = *mean*(*S*)/*std*(*S*), where S is the mean voxel-wise signal within the tissue of interest. The contrast-to-noise ratio (CNR) was calculated between WM and CSF, and between GM and WM, as $CNR_{1/2}=\frac{\left|mean\left(S_{1}\right)-mean\left(S_{2}\right)\right|}{\sqrt{std{\left(S_{1}\right)}^{2}+std{\left(S_{2}\right)}^{2}}}$, where the subscripts 1 and 2 indicate the respective tissue types.

Image quality metrics were computed separately for the RMS images of each repetition using two sets of echo combinations: (i) all available echoes (four for images with navigators and five for images without) and (ii) the first three echoes only. $CNR_{WM/CSF}$ was assessed on images combining all available echoes, as previous work showed that $CNR_{WM/CSF}$ and subjective SC segmentability in ME-GRE images are highest when using all echoes with echo times spanning 7-23 ms (Büeler et al., 2022). $CNR_{GM/WM}$ was assessed on images generated from the first three echoes, based on the same study, which found that $CNR_{GM/WM}$ and subjective GM segmentability are highest when using three echoes with echo times spanning 7-15 ms.

Calculations were performed on a slice-by-slice basis and then averaged across the two slices of the slab. In the lumbosacral cord, where only a single slab was acquired (Fig. 1E), two neuroanatomical landmarks were used (Büeler et al., 2022, 2024): the “LSE slice”, defined as the slice with the largest cross-sectional spinal cord area, and the “CM slice”, defined as the most caudal slice in which GM still exhibits the characteristic butterfly shape. Four pairs of slices were considered: (i) the LSE slice and the slice immediately caudal to it (“LSE”), (ii) the two slices rostral to the two LSE slices (“LSE+1”), (iii) the two slices caudal to the two LSE slices (“LSE-1”), and (iv) the CM slice and the slice immediately caudal to it (“CM”). Quantitative metrics were then averaged across the three repetitions.

Statistical analyses were performed in R (v4.3.2). ME-GRE acquisitions with and without navigators were compared across the cervical, thoracic, and lumbosacral cord (*matched-TR* comparison; *n* = 6) and separately across the lumbosacral cord (*minimum-TR* comparison; *n* = 10). For both comparisons, linear mixed-effects models (*lme4* package, v1.1.37) were fitted to SNR and CNR values, with acquisition type, spinal cord region (cervical, thoracic, and lumbosacral) and their interaction as fixed effects, and participants as random effects. Post-hoc tests comparing image metrics within each region were performed using the *emmeans* package (v1.11.1).

### 2.6 Segmentability ratings

In each participant, images acquired with and without navigators (averaged across three repetitions) were evaluated for segmentability by six experienced raters (S.B., L.B., C.W.K., M.D.L., S.J.V., G.D.). SC segmentability was assessed on images generated by combining all available echoes (four for the images acquired with and five for the images without navigators). GM segmentability was assessed on images generated by combining the first three echoes. Because differences in image reconstruction could introduce scaling discrepancies between images acquired with and without navigators, potentially affecting qualitative ratings, the intensities of the images with navigators were linearly rescaled on a slice-wise basis to match the mean and standard deviation of the corresponding images without navigators (the “reference images”). Specifically, for each slice, the voxel intensities of the image with navigators were first standardized by subtracting their mean and dividing by their standard deviation. The standardized values were then scaled to the reference image by multiplying by its standard deviation and adding its mean.

Raters were presented with pairs of images from the same anatomical location (one acquired with navigators and one without), in a randomized order, blinded to acquisition type. They assigned a segmentability rating to indicate whether the first image was *much better* (+2), *somewhat better* (+1), *no difference* (0), *somewhat worse* (–1), or *much worse* (–2) in terms of SC or GM segmentability. For each participant, ratings were performed on the more caudal slice of each cervical and thoracic slab (seven slice each) and on the more rostral slice of lumbosacral segment (“LSE+1”, “LSE”, “LSE–1”, and “CM”), resulting in 18 slices in total. Each rater provided 36 ratings per participant (two segmentation types × 18 slices). Ratings were then averaged across participants and raters to obtain a mean rating per slice and segmentation type.

### 2.7 Automatic segmentation

Automatic segmentation of the SC and GM was performed using contrast-agnostic, deep learning-based algorithms (Bédard et al., 2025) via *sct_deepseg spinalcord* (Naga Karthik et al., 2026) and *sct_deepseg graymatter* (Medina et al., 2025), respectively, implemented in the Spinal Cord Toolbox (v7.1; De Leener et al., 2017). The limited superior-inferior coverage (two slices per slab) in the cervical and thoracic cord compromised the performance of the 3D SC segmentation model (i.e., a model that uses volumetric context). Therefore, the slabs were concatenated and segmented using a 2D SC segmentation model that performs slice-by-slice segmentation (*sct_deepseg_sc –kernel 2d*). Additionally, SC and GM segmentation was not performed in the conus medullaris, as only a limited amount of data from this region was included in the algorithm’s training set (Naga Karthik et al., 2026). Segmentation was performed on images averaged across three repetitions, using the combination of all available echoes for SC segmentation and the combination of the first three echoes for GM segmentation.

Automatically obtained masks were compared with manually segmented masks, which served as the ground truth (GT). Similarity between masks was evaluated slice-wise using two metrics: (i) cross-sectional area (CSA) and (ii) center of mass (COM). CSA was computed as the number of voxels multiplied by the in-plane voxel area (0.25 mm²). COM was calculated in MATLAB using *regionprops* to extract mask centroids, which were then converted to physical coordinates. COM (in mm) was defined as the Euclidean norm of the centroid position. For each metric, the absolute deviation from the GT was computed for images acquired with and without navigators. Because our primary objective was to evaluate the relative performance of automatic segmentation rather than its absolute accuracy, we calculated the difference in absolute deviations between acquisition types as Δ = |without nav. − GT| − |with nav. − GT|. The main outcome measure was the number of cases in which ΔCSA and ΔCOM were classified as positive or negative outliers, defined as values exceeding the ±3 × interquartile range (IQR). Positive outliers indicate a lower deviation from the GT for images acquired with navigators, whereas negative outliers indicate a lower deviation for images acquired without navigators. Cases in which both acquisition types yielded results equally distant from the ground truth were not classified as outliers.

## 3 Results

### 3.1 Quantitative comparison: Image quality metrics

**Fig. 2.**
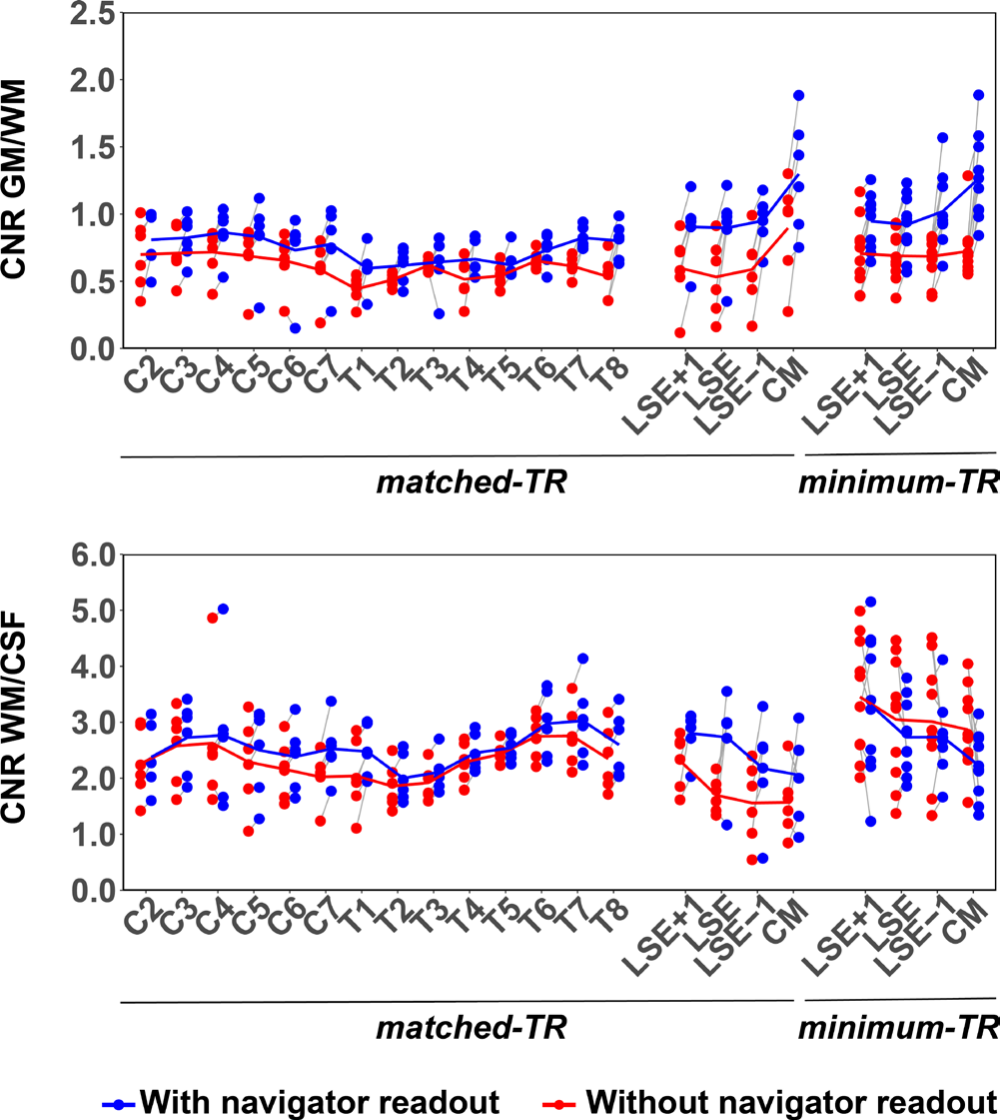
CNR across the spinal cord with and without navigators. Contrast-to-noise ratio (CNR) between gray matter (GM) and white matter (WM), and between WM and cerebrospinal fluid (CSF), measured on multi-echo gradient-echo acquisitions with navigators and optimized navigator processing using a fast Fourier transform-based approach (blue), and on acquisitions without navigators (red). Dots represent individual participants’ values, with values from the same participant connected by a gray line. The two acquisition types were obtained either with matched repetition times (*matched-TR, n* = 6) or with the minimum achievable repetition time (*minimum-TR, n* = 10). Averaged values across participants are shown as a solid line across cervical (C2–C7) and thoracic (T1–T8) vertebral levels, as well as across lumbosacral anatomical landmarks, including the lumbosacral enlargement (LSE), two slices rostral (LSE+1), two slices caudal (LSE-1), and the conus medullaris (CM).

CNR values are presented in Figure 2 for each cervical (C2–C7) and thoracic (T1–T8) vertebral level, as well as for the lumbosacral anatomical landmarks. When comparing sequences with the same TR (*matched-TR* comparison), ANOVA on the linear mixed-effects model revealed significantly higher $CNR_{GM/WM}$ in the ME-GRE images with navigators compared to those without, in the cervical (F(1,55) = 18.61, *p* < 0.001), thoracic (F(1,75) = 39.99, *p* < 0.001), and lumbosacral cord (F(1,35) = 62.31, *p* < 0.001). Notably, in the lumbosacral cord, all participants showed higher values in the navigator-corrected images. For $CNR_{WM/CSF}$ , values were higher in the thoracic (F(1,75) = 6.81, *p* = 0.011) and lumbosacral cord (F(1,35) = 15.07, *p* < 0.001), whereas no significant difference was found in the cervical cord (F(1,55) = 2.19, *p* = 0.144). Post-hoc results for individual spinal levels, including means and standard deviations, are reported in Supplementary Table S1. The combination of the first three echoes (instead of all five available echoes) for $CNR_{WM/CSF}$ showed lower absolute values but comparable relative differences between images acquired with and without navigators (Supplementary Fig. S1).

Navigator-corrected images (using all available echoes) yielded significantly higher $SNR_{GM}$ in the cervical (F(1,55) = 16.95, *p* < 0.001), thoracic (F(1,75) = 36.35, *p* < 0.001), and lumbosacral cord (F(1,35) = 41.08, *p* < 0.001) compared to images acquired without navigators. Similar improvements were observed for $SNR_{WM}$ in the cervical (F(1,55) = 12.38, *p* = 0.001), thoracic (F(1,75) = 51.16, *p* < 0.001), and lumbosacral cord (F(1,35) = 60.78, *p* < 0.001). Post-hoc results for individual spinal levels are summarized in Supplementary Figure S2 and Supplementary Table S2 (combination of the first three echoes) and Supplementary Figure S3 and Supplementary Table S3 (combination of all available echoes).

In the *minimum-TR* comparison in the lumbosacral cord, $\;CNR_{GM/WM}$ (F(1,63) = 92.37, *p* < 0.001), $SNR_{GM}$ (F(1,63) = 56.82, *p* < 0.001), and $SNR_{WM}$ (F(1,63) = 36.04, *p* < 0.001) were again significantly higher in navigator-corrected images, whereas $CNR_{WM/CSF}$ did not differ significantly (F(1,63) = 3.73, *p* = 0.058). Post-hoc tests are provided in Supplementary Tables S1 (CNR) and S2-S3 (SNR).

### 3.2 Qualitative comparison: Segmentability ratings

**Fig. 3.**
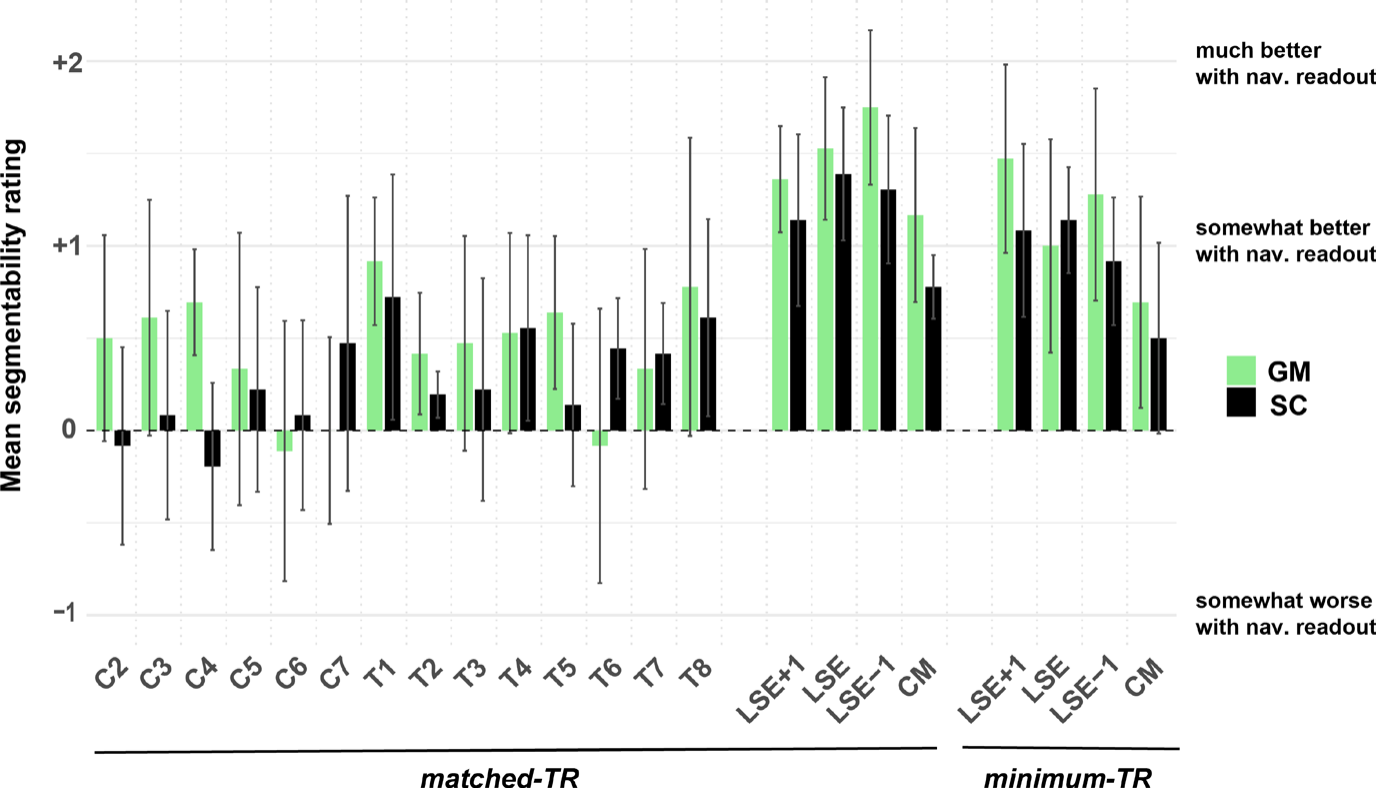
Qualitative comparison. Segmentability ratings of the spinal cord (SC, black) and gray matter (GM, light green) on multi-echo gradient-echo acquisitions with optimized navigator processing versus without navigators, across cervical (C2-C7) and thoracic (T1-T8) vertebral levels, as well as at lumbosacral landmarks: the lumbosacral enlargement (LSE), two slices rostral (LSE+1) and two slices caudal (LSE-1), and the conus medullaris (CM). Values were averaged across participants and raters, and the standard deviation was calculated across raters. Positive values indicate preference for the image with navigators, while negative values indicate preference for the image without. Ratings reflect the degree of preference: the image with navigators is *much better* (+2), *somewhat better* (+1), *no difference* (0), *somewhat worse* (–1), or *much worse* (–2).

**Fig. 4.**
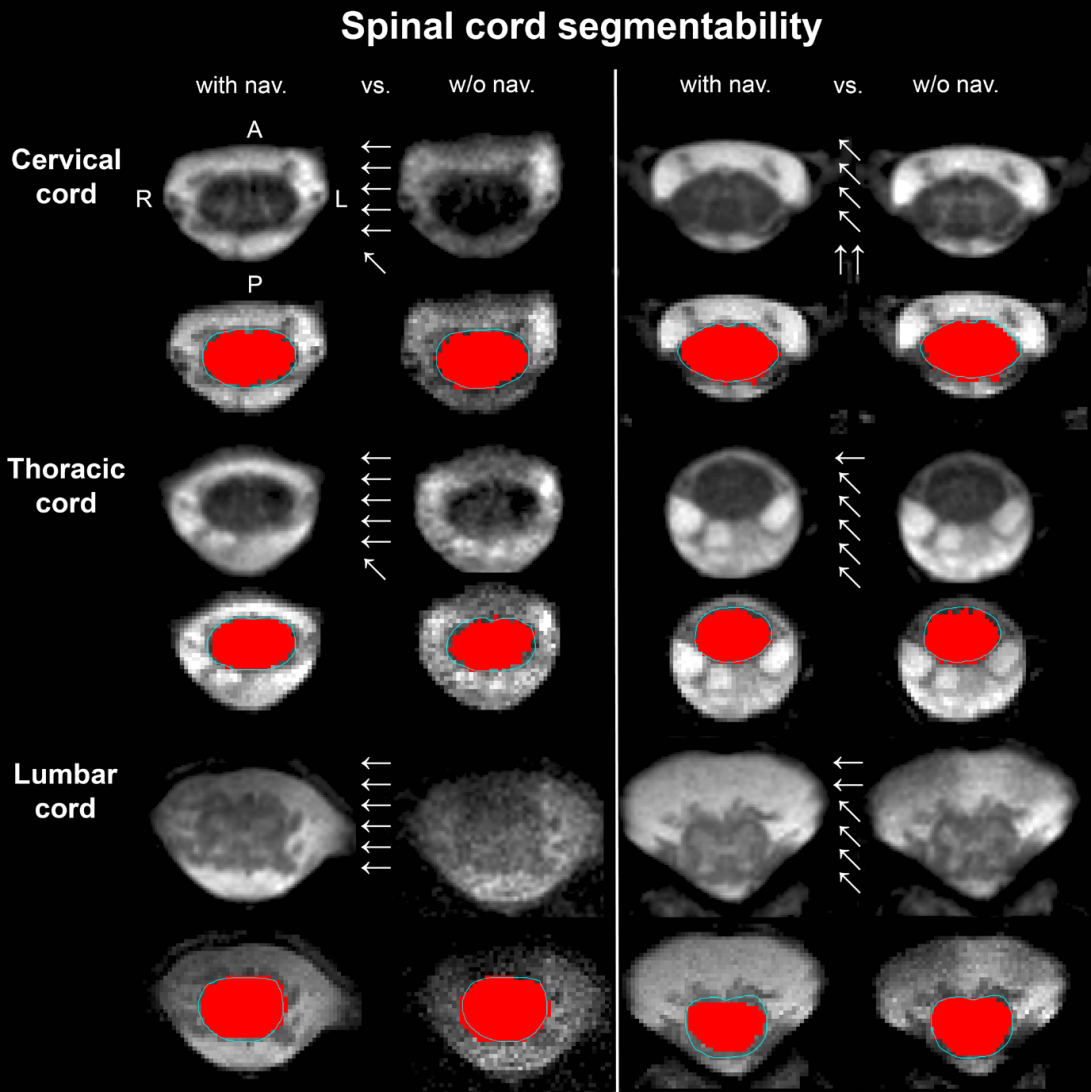
Images illustrating rater preferences and automatic segmentation of spinal cord. Slices of multi-echo gradient-echo acquisitions with navigators and optimized navigator processing, as well as without navigators are shown (*matched-TR* comparison). Example images correspond to slices rated as *much better* (left panel) and *somewhat better* (right panel) by the majority of raters; images are displayed after interpolation for qualitative assessment. Rater preferences are indicated by arrows (← = with navigator much better, ⬉ = somewhat better, ↑ = no difference). Spinal cord masks from the automatic segmentation algorithm (in red; *sct_deepseg_sc* for the cervical and thoracic cord; *sct_deepseg spinalcord* for the lumbar cord) are displayed along with the manual ground-truth segmentation (light blue outline) on images without interpolation. While raters generally preferred images with navigators in terms of segmentability, automatic spinal cord segmentation yielded reasonable results for both acquisition types.

Figure 3 shows segmentability ratings for the SC and GM, averaged across raters and participants. Overall, raters preferred navigator-corrected images across nearly all spinal levels for both SC and GM segmentability. Examples are shown in Figures 4 (SC segmentability) and 5 (GM segmentability). The strongest qualitative improvements were observed in the lumbosacral cord, where 86% and 75% of slices were rated as better (*much better* or *somewhat better*) for GM and SC segmentability, respectively, and only 5% and 4% were rated as worse; no slices were rated as much worse. Improvements were less consistent in the cervical and thoracic cord, with 54% (GM) and 36% (SC) of slices rated as better and 17% and 14% rated as worse. Across most spinal levels, subjective improvements were more pronounced for GM than for SC. In the *minimum-TR* comparison, raters also preferred navigator-corrected images, although segmentability ratings were slightly lower than for the *matched-TR* comparison (Fig. 3).

**Fig. 5.**
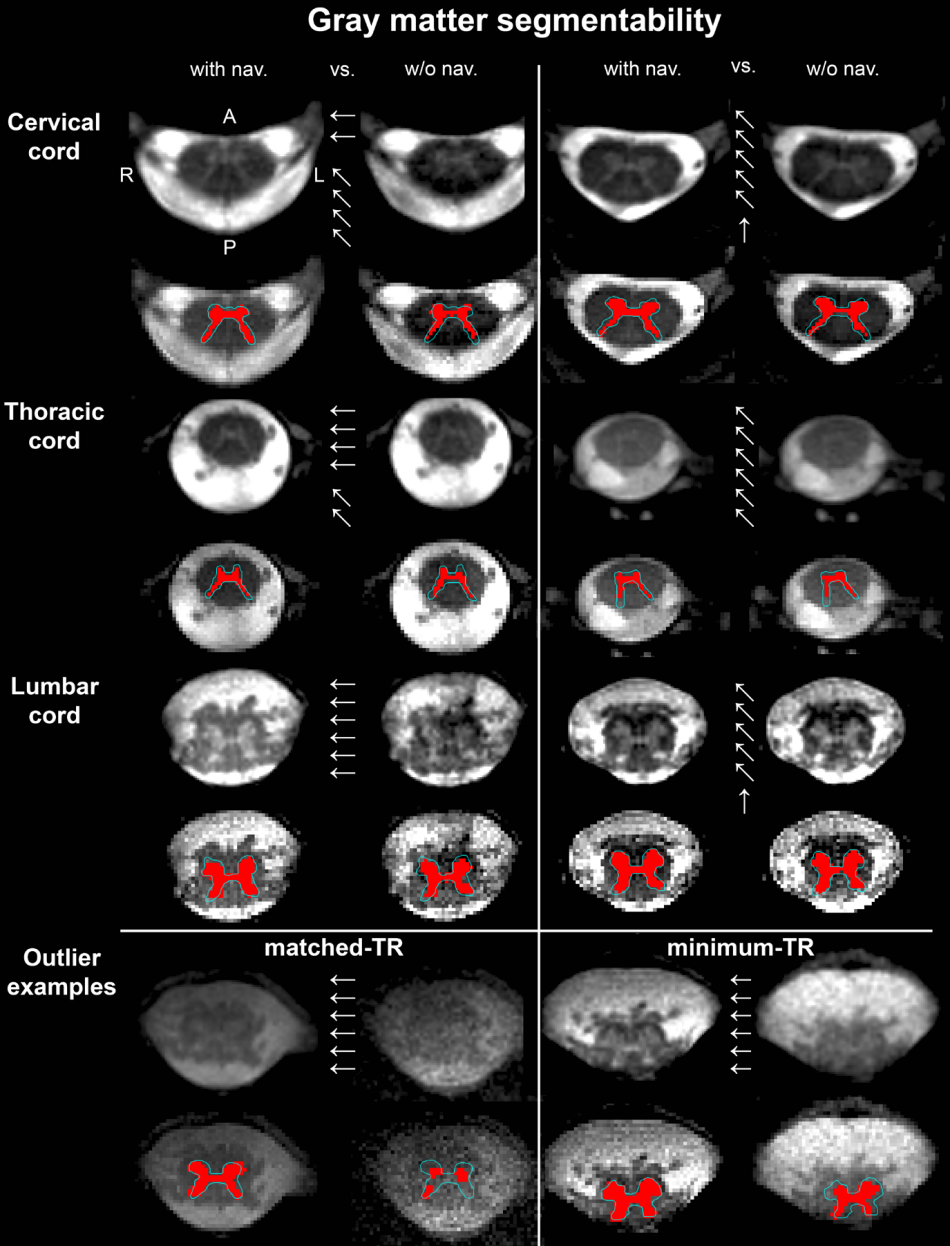
Images illustrating rater preferences and automatic segmentation of gray matter. Slices of multi-echo gradient-echo acquisitions with navigators and optimized navigator processing, as well as without navigators are shown (*matched-TR* comparison). Example images correspond to slices rated as *much better* and *somewhat better* by the majority of raters; images are displayed after interpolation for qualitative assessment. Rater preferences are indicated by arrows (← = with navigator much better, ⬉ = somewhat better, ↑ = no difference). Gray matter masks from the automatic segmentation algorithm (in red, *sct_deepseg graymatter*) are displayed along with the manual ground-truth segmentation (light blue outline) on images without interpolation. Raters generally preferred images acquired with navigators in terms of segmentability. In cases with severe artifacts, most commonly in the lumbosacral cord (see bottom panel “Outlier examples”), automatic segmentation performed better on images acquired with navigators than on those without.

### 3.3 Comparative performance of automatic segmentation

Figures 4 and 5 show examples of automatic SC and GM segmentations, respectively. Supplementary Figure S4 illustrates the differences in absolute deviations (Δ) from the ground truth for CSA and COM between images acquired with and without navigators. Automatic SC segmentation performed well across the whole spinal cord indicating comparable performance between acquisition types (Fig. 4). For automatic GM segmentation, performance was similar for both acquisition types in the cervical and thoracic cord (Fig. 5). In the lumbar cord, large positive outliers were observed for ΔCSA and ΔCOM, indicating that segmentation masks from images with navigators were substantially closer to the ground truth than those from images without navigators (Supplementary Fig. S4). Visual inspection revealed that these cases were affected by severe ghosting artefacts, leading to inaccurate GM segmentation in images without navigators, while images with navigators yielded reasonable segmentations (see bottom row of Fig. 5).

## 4 Discussion

In this study, we investigated whether the recently developed navigator processing pipeline, optimized to correct for breathing-induced B_0_ field fluctuations in the spinal cord, improves the quantitative and qualitative aspects of ME-GRE images in the cervical, thoracic, and lumbosacral cord as compared to standard vendor-provided sequences without navigators. Optimized navigator-based correction reduced artifact load and improved image quality across all spinal levels. Significant increases in CNR between GM and WM were observed near the upper (C7-T1) and lower (T7-T8) borders of the lungs, as well as throughout the lumbosacral cord. In addition, raters preferred images acquired with navigators and optimized navigator processing over those acquired without, with the most pronounced benefit observed for GM segmentability in the lumbosacral cord.

### 4.1 Spinal cord and gray matter segmentability improves with optimized navigator processing

Cross-sectional area of the SC, GM, and WM has been widely used as a proxy for tissue atrophy in spinal cord disorders such as spinal cord injury (David et al., 2021) and amyotrophic lateral sclerosis (Paquin et al., 2018). Accurate segmentation is essential for these measurements. While high CNR between GM and WM (for GM segmentation) and between WM and CSF (for SC segmentation) is important, other image properties, including boundary sharpness, signal homogeneity, and artifact load, also strongly influence perceived segmentability (Wang et al., 2016).

Navigator-corrected images showed higher CNR between GM and WM and between WM and CSF at the cervical-thoracic junction (C7-T1), in the thoracic cord (T7-T8), and across the lumbosacral cord. These regions have previously been identified as exhibiting the largest B_0_ field fluctuations (Beghini et al., 2025). Blinded qualitative ratings supported these findings: raters preferred navigator-corrected images, with the strongest preference observed in the lumbosacral cord, followed by the thoracic and cervical cord. Specifically, for GM segmentability in the lumbosacral cord, 42% of slices were rated as *much better* and 44% *somewhat better* when acquired with navigators, followed by the thoracic (7% *much better*, 50% *somewhat better*) and the cervical cord (8% *much better*, 42% *somewhat better*). Raters attributed their preferences to sharper SC and GM boundaries, clearer visualization of dorsal and ventral rootlets, and more distinct dorsal GM horns, features that were most evident in the navigator-reconstructed images (Figs. 4-5).

For most spinal levels, rater-assessed improvements in segmentability were more pronounced for GM. We argue that, because the $CNR_{WM/CSF}$ is substantially higher than $CNR_{GM/WM}$ , further increases yield smaller qualitative gains for SC segmentability. Moreover, owing to the simpler geometry of the SC compared with GM, SC segmentation is likely to tolerate lower image quality. We note that qualitative improvements were evaluated on images averaged across three repetitions; therefore, improvements in individual acquisitions are likely to be greater (Beghini et al., 2025). Furthermore, although not acquired here, navigator-based reconstruction is expected to have an even greater impact at the T9-T10 levels, which are likely to exhibit the largest B_0_ field fluctuations (Beghini et al., 2025). Caudal to lower lung border, in the lumbosacral cord, strong breathing-induced ghosting was observed in 10% (1/10) of participants in the present study. Upon retrospective review of the images from our previous study, a comparable proportion of strong breathing-induced artefacts was observed, with 10% (5/48) of participants exhibiting pronounced ghosting at this level (Büeler et al., 2025).

While matching TR between sequences is ideal for isolating navigator effects, clinical priorities often favor minimizing TR and applying standard image filters. To examine this, we compared images acquired with navigators with standard ME-GRE images acquired at the shortest possible TR with standard filtering (2D distortion correction, coil sensitivity correction, and an elliptical filter) in the lumbosacral cord (*minimum-TR* comparison). Under these conditions, $CNR_{WM/CSF}$ no longer differed significantly between the two methods, likely because coil sensitivity correction disproportionately increased CSF signal anterior to the spinal cord, where it is farther from the spine coils. Nevertheless, raters still preferred navigator images for SC segmentability, indicating that qualitative assessments are influenced by other factors such as artifact load and tissue uniformity, which are less affected by the filters. $CNR_{GM/WM}$ and GM segmentability ratings were not impacted, likely reflecting that the slightly shorter TR (878 ms vs. 899 ms) has only minimal effect on image contrast, and that the applied filters exert only a minor influence on the appearance of the GM-WM interface.

Overall, navigator-corrected images improved both GM and SC segmentability. Across most spinal levels, the improvement was more pronounced for GM. Because GM-WM contrast is substantially lower than WM-CSF contrast, improvements in CNR are more readily reflected in qualitative ratings for GM.

### 4.2 Optimized navigator processing benefits automatic gray matter segmentation

To investigate whether improvements in quantitative and qualitative image metrics translate into improved automatic SC and GM segmentation, we applied state-of-the-art deep learning-based algorithms from the Spinal Cord Toolbox. Importantly, our primary goal was not to benchmark segmentation performance per se, but to compare performance between acquisition types. In particular, we focused on cases in which deviations from manual segmentation (the ground truth) were substantially reduced in navigator-corrected images.

For automatic SC segmentation, we observed no major differences between acquisition types. This indicates that, despite lower image quality and reduced subjective segmentability ratings in images without navigators, the SC segmentation algorithm remains robust, as the SC is still clearly visible (an example is shown in Fig. 4). While the automatic segmentation analysis assessed only success versus failure, subjective segmentability ratings reflect the rater’s confidence. This explains why navigator-corrected images received higher ratings in the lumbar cord, even though automatic SC segmentation succeeded for both acquisition types (Fig. 4). A natural next step would be to evaluate the test-retest reliability of segmentations, which we anticipate would be higher for both manual and automatic segmentations on navigator-corrected images.

For automatic GM segmentation, performance was comparable between acquisition types in the cervical and thoracic cord. In lumbar-cord slices affected by severe artifacts, automatic GM segmentation failed on images without navigators but produced reasonable results on navigator-corrected images (Fig. 5, *matched-TR*). This is expected, as the GM is often not clearly visible without navigators, limiting the algorithm’s ability to generate accurate segmentations. When applying the shortest possible TR and standard image filters on images without navigators (*minimum-TR* comparison), the performance of the automatic GM segmentation improved, also in slices affected by severe artifacts (Fig. 5, minimum-TR). Nevertheless, quantitative image metrics (Fig. 2) and raters’ confidence favored navigator-corrected images, as reflected by the GM segmentability rating (Fig. 3).

Overall, these findings indicate that improvements in image quality achieved through navigator echoes and optimized navigator processing enhance automatic GM segmentation, particularly in regions prone to artifacts, such as the lumbar cord. In contrast, automatic SC segmentation is relatively robust and less dependent on navigator acquisition, as long as the SC remains visible.

### 4.3 Vendor’s navigator reconstruction

For images acquired with navigators (Siemens “Phase stabilization”), we also visually evaluated the images generated using the vendor’s standard navigator processing and reconstruction. We found that for most cervical and thoracic acquisitions, the image quality was visually comparable to that obtained with our custom navigator processing optimized for the spinal cord (fast Fourier transform-based reconstruction) (Beghini et al., 2025) and was generally better or at least equivalent to the images acquired without navigators (Supplementary Fig. S5). However, the vendor’s standard navigator correction failed in cases with severe artifacts, particularly in the lumbosacral cord (Supplementary Fig. S5), underscoring the limitations of the vendor’s correction.

It should be noted that vendor implementations are typically proprietary and not openly documented. In contrast, our Julia-based platform for navigator processing is open-source and highly flexible, allowing full customization. For instance, in cases of more severe field fluctuations, a navigator processing pipeline incorporating an additional phase-unwrapping step has been proposed (Beghini et al., 2025). However, this approach relies on external respiratory belt recordings, which may not always be available in clinical settings.

### 4.4 Limitations

Although the sample size in this study is typical for methodological investigations, a larger and more heterogeneous cohort, including clinical populations, could enable a more comprehensive evaluation of the correction performance. Clinical cohorts may include individuals with higher BMI. However, since higher BMI can lead to larger B_0_ fluctuations (Duerst et al., 2016), the impact of navigator correction may be expected to be even greater in this population. Manual segmentations were derived from navigator-corrected images, as images without navigators were not always segmentable (Fig. 5). Therefore, comparisons of CNR and SNR may be biased toward the navigator images if spatial mismatches existed between sequences with and without navigators. Although slice alignment was performed across repetitions and between acquisition types, sub-voxel shifts (e.g., half-voxel) could not be fully corrected. This limitation was mitigated by using conservative SC and WM masks, which were sufficiently restrictive to remain within the same tissue despite minor misalignments. Notably, the largest differences in CNR and SNR were observed in the lumbosacral cord, where manual adjustments were rarely required.

We note that the current offline reconstruction requires a low-resolution GRE reference scan (acquisition time: 1:04 min) for the parallel imaging reconstruction (SENSE), slightly increasing the total imaging time. In principle, it should be possible to replace this with the reference data acquired for the parallel imaging implementation of the scanner’s built-in image reconstruction, eliminating the need for a dedicated reference scan. Future implementations of the offline reconstruction should investigate this possibility.

### 4.5 Outlook

Clinical applications aiming to quantify SC, GM, and WM atrophy in the spinal cord are likely to benefit from image acquisition techniques incorporating navigators, as these enable more accurate and precise segmentation. Reliable and precise tissue-specific segmentation is particularly important because it can reduce the sample size required to detect a given effect size in clinical trials. The enhanced image quality can improve the performance of automatic segmentation algorithms, as demonstrated for GM segmentation, and may also facilitate shorter scan times by decreasing the number of required averages. Furthermore, applying the optimized navigator correction could improve the reliability of quantitative results, which is especially relevant for longitudinal and multi-center studies where robustness to physiological variability is essential. As the optimized navigator correction currently requires offline image reconstruction, an important future step toward clinical implementation will be the development of an online image reconstruction pipeline.

## 5 Conclusion

Navigator processing optimized for the spinal cord improves the segmentability of both spinal cord and gray matter in multi-echo gradient-echo acquisitions, particularly in regions affected by breathing-induced artifacts, such as the lumbosacral cord. While automatic spinal cord segmentation is generally robust, gray matter segmentation benefits substantially from navigator-based correction, remaining accurate where uncorrected images fail. These findings support optimized navigator-based correction as a valuable approach for reliable cross-sectional tissue measurements throughout the spinal cord.

## Supplementary Material

Supplementary Material

## Data Availability

The data underlying the results presented in the study are available on GitHub (https://github.com/NeuroimagingBalgrist/NavCorr_on_SpinalCord_MEGRE).
