# Supplementary material for: Navigator readouts with optimized processing improve spinal cord and gray matter segmentability in multi-echo gradient-echo imaging
Source: Imaging Neurosci (Camb). 2026 Aug 3;4:IMAG.a.1320. doi: 10.1162/IMAG.a.1320 (PMC13435687; doi:10.1162/IMAG.a.1320)
Supplement: Supplementary file 1 [file IMAG.a.1320_supp.pdf]

Supplementary material

Supplementary Table S1. Contrast-to-noise ratio (CNR) for images acquired with and without navigators across the cervical, thoracic, and lumbosacral cord (first three echoes for CNR GM/WM and all available echoes for CNR WM/CSF). Values represent group mean ± SD.

	Segment	CNR GM/WM				CNR WM/CSF			
		with nav. mean ± SD	w/o nav. mean ± SD	Diff (%)	p-value	with nav. mean ± SD	w/o nav. mean ± SD	Diff (%)	p-value
		matched-TR comparison							
Cervical cord	C2	0.81 ± 0.21	0.70 ± 0.25	15.80	0.141	2.38 ± 0.58	2.26 ± 0.61	5.22	0.751
	C3	0.82 ± 0.16	0.71 ± 0.19	16.08	0.129	2.73 ± 0.64	2.58 ± 0.66	5.83	0.687
	C4	0.86 ± 0.18	0.72 ± 0.17	20.49	0.052	2.77 ± 1.26	2.63 ± 1.15	5.32	0.707
	C5	0.83 ± 0.28	0.69 ± 0.22	20.73	0.059	2.50 ± 0.78	2.29 ± 0.78	9.17	0.573
	C6	0.73 ± 0.29	0.66 ± 0.20	11.63	0.307	2.38 ± 0.57	2.16 ± 0.52	10.30	0.550
	C7	0.78 ± 0.27	0.58 ± 0.21	32.67	0.013	2.53 ± 0.52	2.02 ± 0.43	24.83	0.180
Thoracic cord	T1	0.60 ± 0.16	0.44 ± 0.10	34.99	0.012	2.48 ± 0.45	2.04 ± 0.64	21.39	0.077
	T2	0.62 ± 0.13	0.51 ± 0.05	21.10	0.078	2.00 ± 0.41	1.86 ± 0.40	7.64	0.561
	T3	0.64 ± 0.21	0.62 ± 0.04	4.10	0.675	2.11 ± 0.33	1.92 ± 0.30	9.96	0.435
	T4	0.66 ± 0.12	0.52 ± 0.16	28.91	0.016	2.45 ± 0.33	2.28 ± 0.35	7.34	0.493
	T5	0.62 ± 0.11	0.54 ± 0.09	14.95	0.184	2.54 ± 0.23	2.43 ± 0.20	4.63	0.645
	T6	0.73 ± 0.12	0.65 ± 0.07	12.61	0.176	2.98 ± 0.57	2.75 ± 0.39	8.25	0.355
	T7	0.82 ± 0.08	0.61 ± 0.07	34.36	0.001	3.03 ± 0.68	2.76 ± 0.54	9.78	0.272
	T8	0.80 ± 0.14	0.53 ± 0.16	49.97	<.001	2.60 ± 0.58	2.35 ± 0.57	10.69	0.306
Lumbosacral cord	LSE+1	0.90 ± 0.24	0.60 ± 0.27	51.19	0.002	2.80 ± 0.40	2.31 ± 0.48	21.23	0.156
	LSE	0.90 ± 0.29	0.53 ± 0.28	68.47	<.001	2.73 ± 0.82	1.70 ± 0.31	60.83	0.004
	LSE-1	0.95 ± 0.18	0.59 ± 0.28	61.76	<.001	2.17 ± 0.91	1.56 ± 0.70	39.57	0.077
	CM	1.30 ± 0.42	0.90 ± 0.37	44.87	<.001	2.06 ± 0.80	1.57 ± 0.59	31.11	0.158
		minimum-TR comparison							
	LSE+1	0.95 ± 0.20	0.71 ± 0.25	34.22	0.001	3.31 ± 1.24	3.45 ± 1.06	-4.17	0.687
	LSE	0.92 ± 0.22	0.69 ± 0.18	33.47	0.002	2.73 ± 0.65	3.05 ± 1.09	-10.43	0.375
	LSE-1	1.02 ± 0.27	0.68 ± 0.17	48.88	<.001	2.74 ± 0.63	3.01 ± 1.05	-9.12	0.444
	CM	1.26 ± 0.32	0.72 ± 0.22	74.70	<.001	2.23 ± 0.58	2.87 ± 0.74	-22.26	0.078

Abbreviations: CM: Conus medullaris, CSF: cerebrospinal fluid, GM: gray matter, LSE: lumbosacral enlargement, SD: standard deviation, WM: white matter.

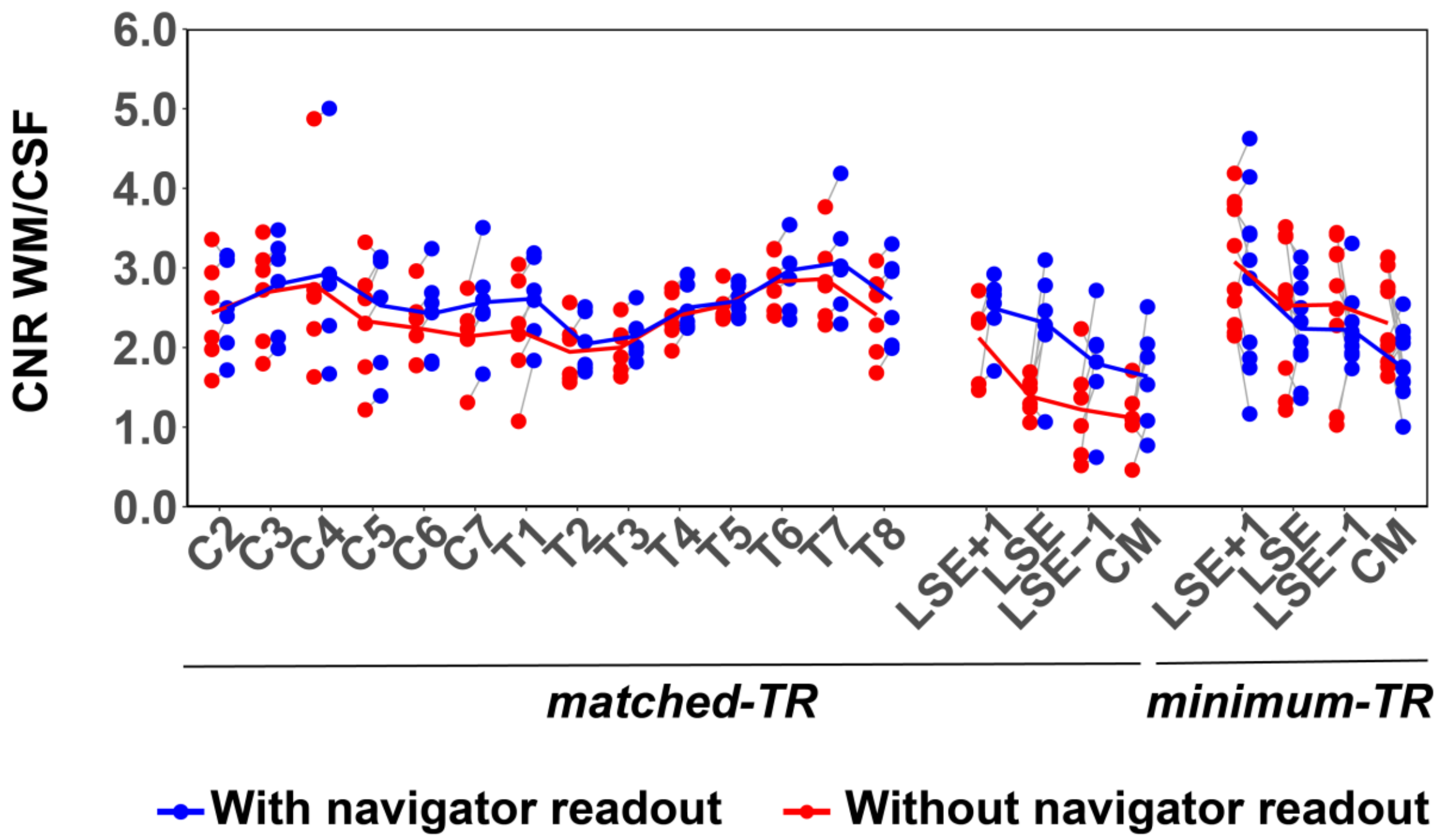

Supplementary Figure S1. CNR across the spinal cord with and without navigators. Contrast-to-noise ratio (CNR) between WM and cerebrospinal fluid (CSF), measured on multi-echo gradient-echo acquisitions (first three echoes) with navigators and optimized navigator processing using a fast Fourier transform-based approach (blue), and on acquisitions without navigators (red). Dots represent individual participants' values, with values from the same participant connected by a gray line. The two acquisition types were obtained either with matched repetition times (*matched-TR, n* = 6) or with the minimum achievable repetition time (*minimum-TR, n* = 10). Averaged values across participants are shown as a solid line across cervical (C2–C7) and thoracic (T1–T8) vertebral levels, as well as across lumbosacral anatomical landmarks, including the lumbosacral enlargement (LSE), two slices rostral (LSE+1), two slices caudal (LSE-1), and the conus medullaris (CM).

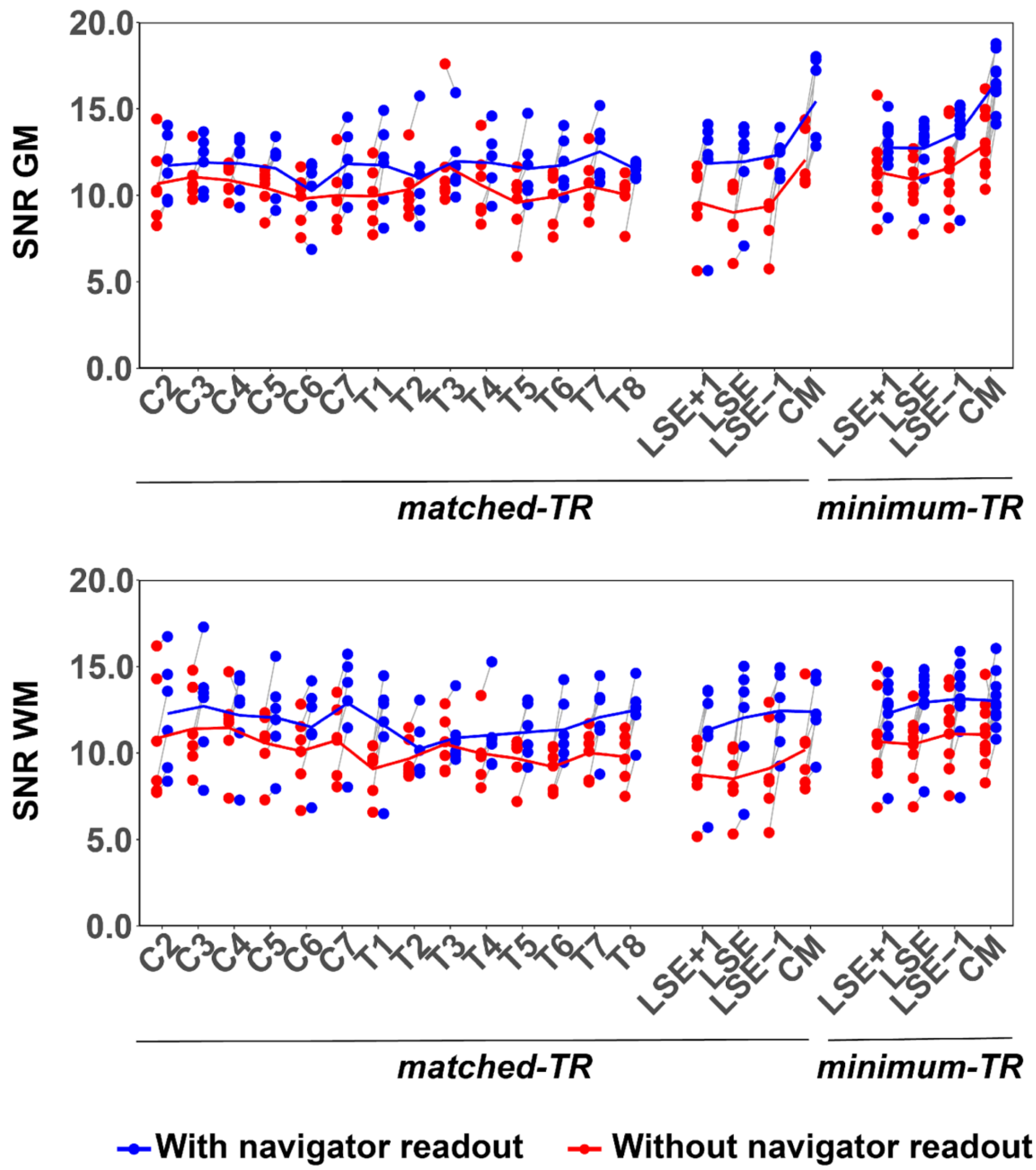

Supplementary Figure S2. SNR across the spinal cord with and without navigators (computed on the first three echoes). Signal-to-noise ratio (SNR) for gray matter (GM) and white matter (WM) measured on multi-echo gradient-echo acquisitions (first three echoes) with navigators and optimized navigator processing using a fast Fourier transform-based approach (blue), and on acquisitions without navigators (red). Dots represent individual participants' values, with values from the same participant connected by a gray line. The two acquisition types were obtained either with matched repetition times (*matched-TR, n* = 6) or with the minimum achievable repetition time (*minimum-TR, n* = 10). Averaged values across participants are shown as a solid line across cervical (C2–C7) and thoracic (T1–T8) vertebral levels, as well as across lumbosacral anatomical landmarks, including the lumbosacral enlargement (LSE), two slices rostral (LSE+1), two slices caudal (LSE-1), and the conus medullaris (CM).

Supplementary Table S2. Signal-to-noise ratio (SNR) values with and without navigators across the cervical, thoracic, and lumbosacral cord (first three echoes). Values represent group mean ± SD.

	Segment	SNR GM with nav. mean ± SD	SNR GM w/o nav. mean ± SD	SNR GM Diff (%)	SNR GM p-value	SNR WM with nav. mean ± SD	SNR WM w/o nav. mean ± SD	SNR WM Diff (%)	SNR WM p-value
		matched-TR comparison							
Cervical cord	C2	12.05 ± 1.51	11.20 ± 2.23	7.61	0.137	12.45 ± 3.12	11.57 ± 3.69	7.59	0.344
	C3	12.17 ± 1.34	11.68 ± 1.32	4.19	0.389	13.00 ± 3.14	12.09 ± 2.56	7.52	0.327
	C4	12.51 ± 1.76	11.25 ± 0.89	11.16	0.030	12.93 ± 3.39	12.05 ± 2.41	7.28	0.344
	C5	11.57 ± 1.70	10.59 ± 1.45	9.17	0.090	12.04 ± 2.41	10.89 ± 1.82	10.52	0.218
	C6	10.56 ± 2.05	10.25 ± 1.76	2.97	0.591	11.67 ± 2.55	10.62 ± 2.43	9.91	0.258
	C7	11.93 ± 2.02	10.22 ± 1.85	16.75	0.004	12.86 ± 2.68	11.00 ± 2.09	16.94	0.048
Thoracic cord	T1	11.69 ± 2.39	9.81 ± 2.14	19.10	0.004	11.80 ± 2.98	9.39 ± 2.15	25.74	0.001
	T2	11.10 ± 2.65	10.33 ± 1.74	7.46	0.222	10.16 ± 1.88	9.80 ± 1.21	3.71	0.588
	T3	11.60 ± 2.11	11.03 ± 2.57	5.17	0.364	10.64 ± 1.58	10.25 ± 1.60	3.82	0.560
	T4	11.67 ± 1.88	10.24 ± 1.96	14.03	0.024	10.71 ± 1.94	9.88 ± 1.87	8.41	0.218
	T5	11.38 ± 1.97	9.51 ± 1.82	19.57	0.004	11.03 ± 1.57	9.66 ± 1.39	14.24	0.043
	T6	11.71 ± 1.66	9.85 ± 1.56	18.91	0.004	11.24 ± 1.82	9.50 ± 1.46	18.35	0.011
	T7	12.29 ± 2.14	10.35 ± 1.84	18.78	0.003	11.96 ± 2.08	9.77 ± 1.39	22.44	0.002
	T8	11.47 ± 0.72	9.88 ± 1.35	16.13	0.013	11.79 ± 1.66	9.70 ± 1.49	21.59	0.003
Lumbosacral cord	LSE+1	11.77 ± 3.34	9.32 ± 2.40	26.32	0.009	11.14 ± 3.15	8.60 ± 2.23	29.56	0.002
	LSE	11.84 ± 2.61	8.67 ± 1.95	36.53	0.001	11.58 ± 3.14	8.11 ± 1.92	42.74	<.001
	LSE-1	12.19 ± 1.05	9.02 ± 2.48	35.18	0.001	12.14 ± 1.98	8.74 ± 2.82	38.78	<.001
	CM	15.10 ± 2.16	11.35 ± 1.26	33.07	<.001	12.41 ± 1.74	9.79 ± 2.29	26.78	0.001
		minimum-TR comparison							
	LSE+1	12.23 ± 1.98	11.17 ± 2.22	9.45	0.067	11.79 ± 2.27	10.48 ± 2.31	12.44	0.022
	LSE	12.29 ± 1.79	10.90 ± 1.62	12.73	0.017	12.44 ± 1.92	10.52 ± 2.02	18.30	0.001
	LSE-1	13.16 ± 1.86	11.26 ± 2.06	16.92	0.001	12.52 ± 2.25	10.87 ± 2.07	15.17	0.004
	CM	15.68 ± 1.28	12.91 ± 1.95	21.46	<.001	12.68 ± 1.38	11.32 ± 1.45	11.99	0.017

Abbreviations: CM: conus medullaris, GM: gray matter, LSE: lumbosacral enlargement, nav.: navigators, SD: standard deviation, WM: white matter.

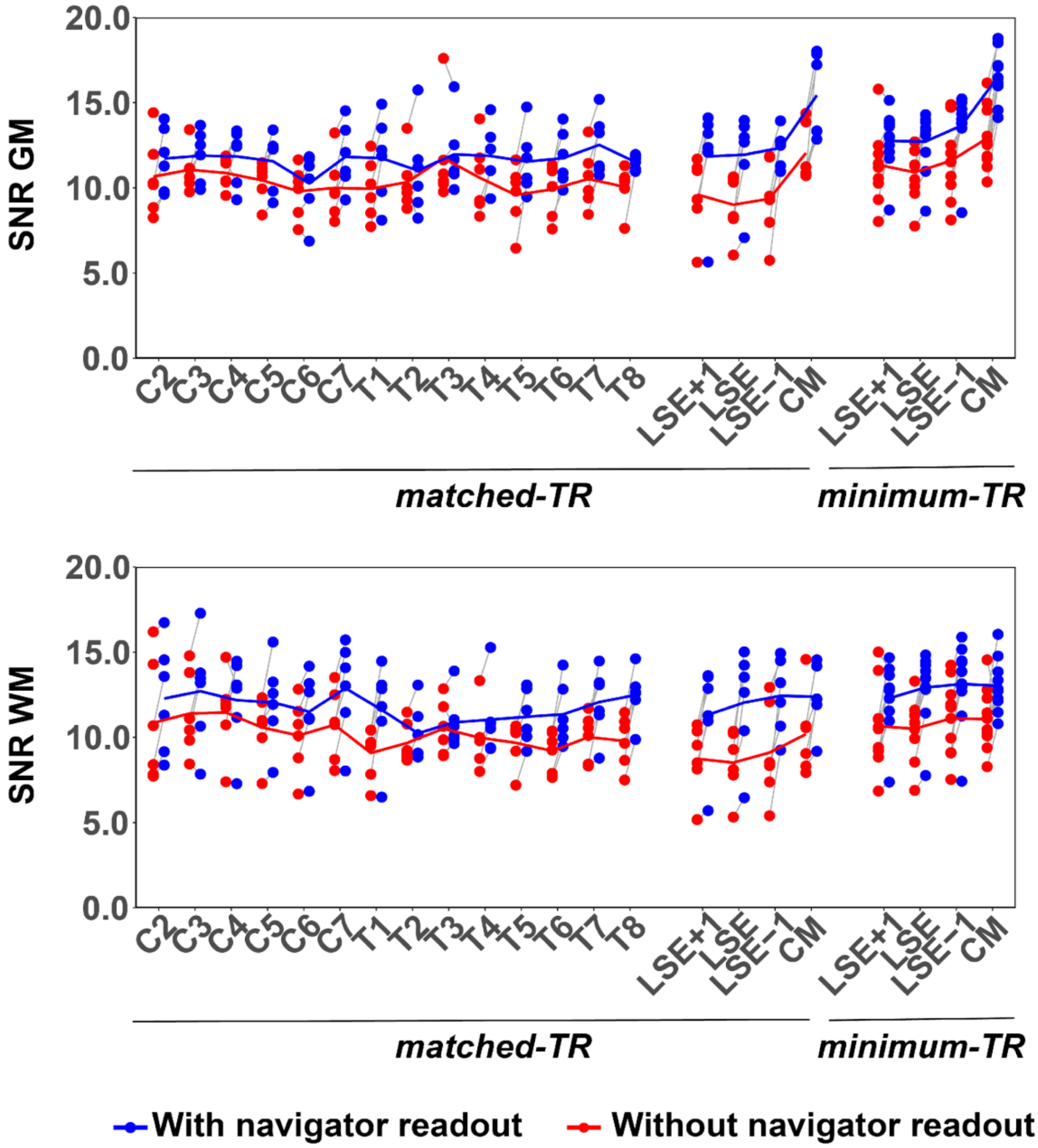

Supplementary Figure S3. SNR across the spinal cord with and without navigators (computed on all available echoes). Signal-to-noise ratio (SNR) for gray matter (GM) and white matter (WM) measured on multi-echo gradient-echo acquisitions (all available echoes) with navigators and optimized navigator processing using a fast Fourier transform-based approach (blue), and on acquisitions without navigators (red). Dots represent individual participants' values, with values from the same participant connected by a gray line. The two acquisition types were obtained either with matched repetition times (*matched-TR, n* = 6) or with the minimum achievable repetition time (*minimum-TR, n* = 10). Averaged values across participants are shown as a solid line across cervical (C2–C7) and thoracic (T1–T8) vertebral levels, as well as across lumbosacral anatomical landmarks, including the lumbosacral enlargement (LSE), two slices rostral (LSE+1), two slices caudal (LSE-1), and the conus medullaris (CM).

Supplementary Table S3. Signal-to-noise ratio (SNR) values with and without navigators across the cervical, thoracic, and lumbosacral cord (all available echoes). Values represent group mean ± SD.

	Segment	SNR GM				SNR WM			
		with nav. mean ± SD	w/o nav. mean ± SD	Diff (%)	p-value	with nav. mean ± SD	w/o nav. mean ± SD	Diff (%)	p-value
		matched-TR comparison							
Cervical cord	C2	11.72 ± 1.85	10.66 ± 2.24	9.92	0.094	12.28 ± 3.24	10.86 ± 3.61	13.13	0.152
	C3	11.89 ± 1.53	11.06 ± 1.27	7.51	0.187	12.71 ± 3.19	11.40 ± 2.43	11.47	0.189
	C4	11.84 ± 1.65	10.87 ± 0.90	8.93	0.124	12.18 ± 2.67	11.46 ± 2.39	6.31	0.465
	C5	11.56 ± 1.69	10.44 ± 1.12	10.71	0.077	12.04 ± 2.54	10.58 ± 1.82	13.83	0.142
	C6	10.26 ± 1.89	9.80 ± 1.50	4.71	0.461	11.51 ± 2.58	10.11 ± 2.16	13.77	0.162
	C7	11.82 ± 1.91	10.00 ± 1.84	18.25	0.005	12.88 ± 2.81	10.73 ± 2.11	20.03	0.033
Thoracic cord	T1	11.73 ± 2.47	9.94 ± 1.75	18.02	0.008	11.61 ± 2.77	9.08 ± 1.55	27.80	<.001
	T2	11.02 ± 2.64	10.33 ± 1.68	6.61	0.303	10.24 ± 1.65	9.67 ± 1.17	5.85	0.380
	T3	11.97 ± 2.13	11.75 ± 2.94	1.90	0.736	10.88 ± 1.56	10.52 ± 1.57	3.49	0.568
	T4	11.87 ± 1.82	10.60 ± 2.13	12.04	0.056	11.04 ± 2.19	9.98 ± 1.82	10.65	0.101
	T5	11.53 ± 1.89	9.58 ± 1.82	20.43	0.004	11.21 ± 1.57	9.68 ± 1.32	15.75	0.020
	T6	11.74 ± 1.61	9.91 ± 1.59	18.44	0.007	11.35 ± 1.83	9.12 ± 1.18	23.38	0.001
	T7	12.52 ± 1.76	10.52 ± 1.71	18.97	0.003	12.07 ± 1.99	10.01 ± 1.37	20.58	0.002
	T8	11.51 ± 0.34	10.05 ± 1.27	14.49	0.030	12.48 ± 1.53	9.79 ± 1.50	27.52	<.001
Lumbosacral cord	LSE+1	11.85 ± 3.13	9.61 ± 2.25	23.31	0.018	11.33 ± 2.98	8.74 ± 2.02	29.58	0.001
	LSE	11.94 ± 2.55	9.01 ± 1.82	32.58	0.003	12.04 ± 3.17	8.52 ± 1.89	41.44	<.001
	LSE-1	12.35 ± 1.08	9.37 ± 2.33	31.84	0.002	12.44 ± 2.21	9.11 ± 2.87	36.50	<.001
	CM	15.43 ± 2.50	12.05 ± 1.63	28.10	0.001	12.39 ± 1.92	10.19 ± 2.43	21.63	0.006
		minimum-TR comparison							
	LSE+1	12.75 ± 1.73	11.35 ± 2.10	12.30	0.021	12.29 ± 2.05	10.65 ± 2.39	15.42	0.017
	LSE	12.72 ± 1.74	10.91 ± 1.46	16.62	0.003	12.93 ± 2.06	10.50 ± 1.75	23.11	0.001
	LSE-1	13.66 ± 1.89	11.58 ± 2.18	17.95	0.001	13.13 ± 2.40	11.11 ± 2.18	18.18	0.004
	CM	16.53 ± 1.48	12.91 ± 1.81	28.00	<.001	13.03 ± 1.56	11.07 ± 1.81	17.70	0.005

Abbreviations: CM: Conus medullaris, GM: gray matter, LSE: lumbosacral enlargement, SD: standard deviation, WM: white matter.

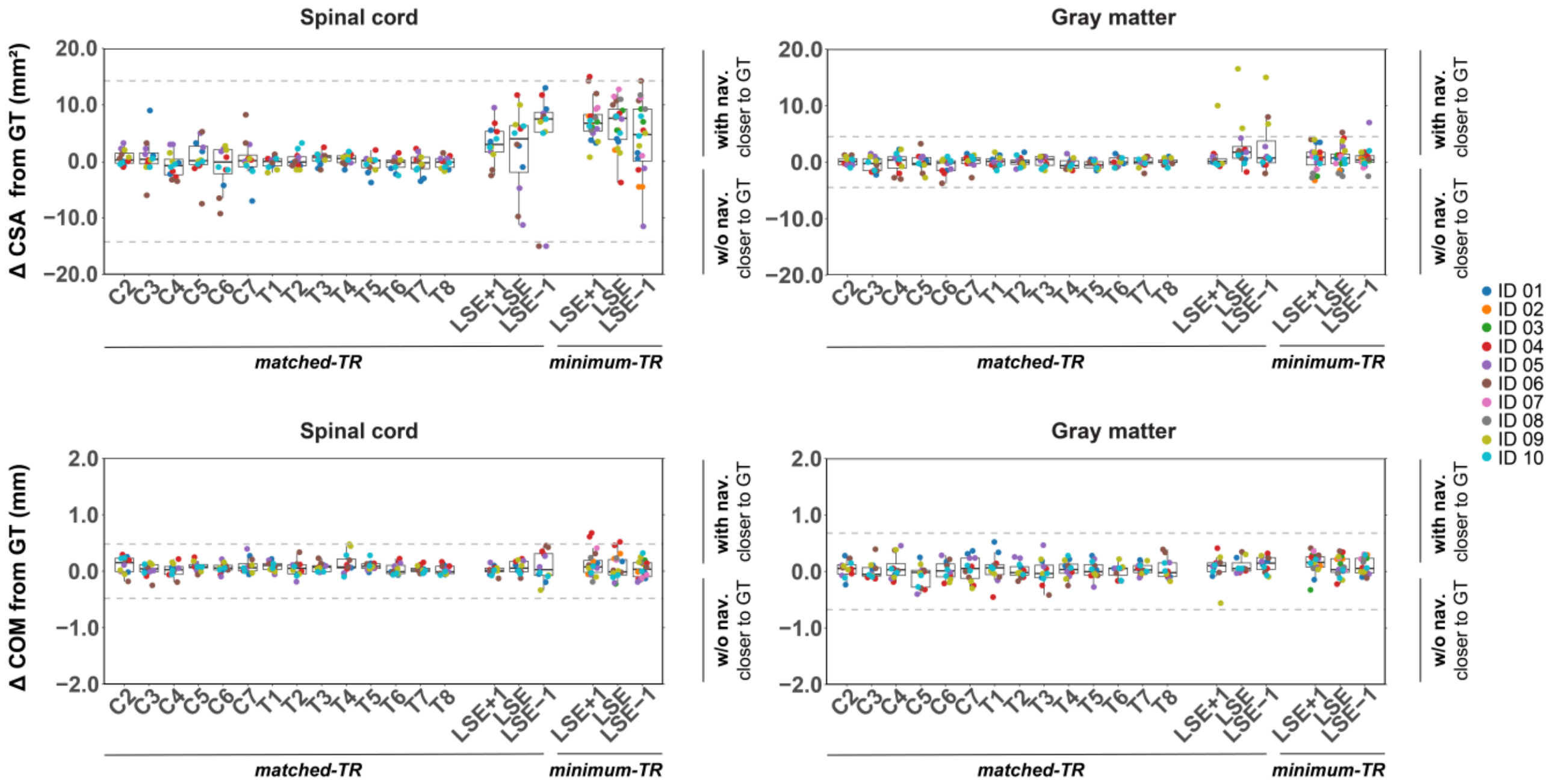

Supplementary Figure S4. Comparative performance of automatic segmentation. Box plots show the differences in absolute deviations (Δ) from the ground truth (GT) between images acquired with and without navigators, for cross-sectional area (CSA) and center of mass (COM). Positive values indicate that masks from images with navigators are closer to the GT, while negative values indicate that masks from images without navigators are closer. Dotted horizontal lines represent ±3 × the interquartile range. One slice for ΔCOM in GM segmentation is missing because segmentation could not be performed on the image acquired without navigators (LSE, matched-TR, ID 09).

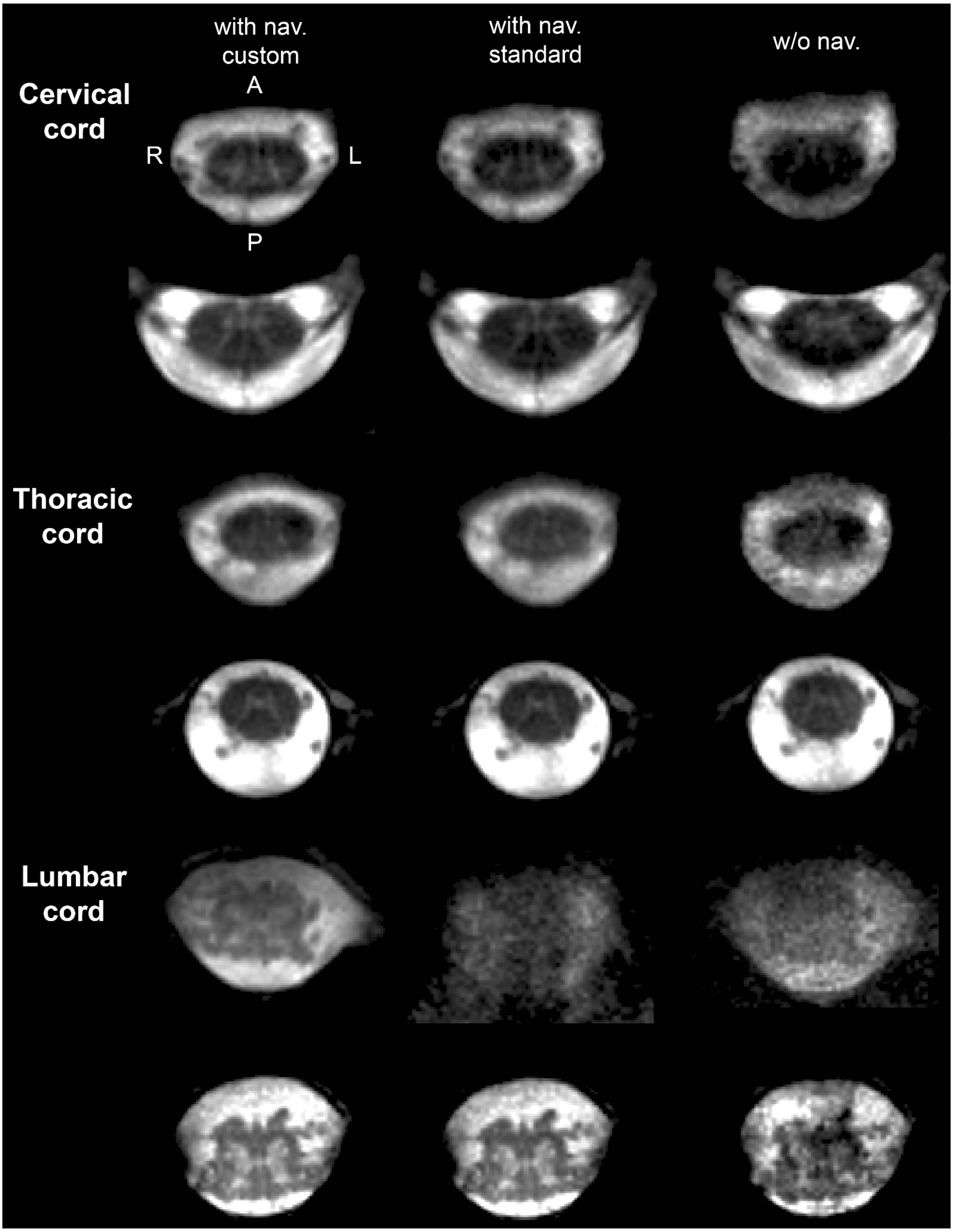

Supplementary Figure S5. Illustration of vendor's navigator reconstruction. Example images acquired with navigators and reconstructed using either our custom navigator processing pipeline (nav. custom) or the standard Siemens method (nav. standard), alongside images acquired without navigators. The slices correspond to those shown on the left side of Fig. 4 and 5.
